# Biomechanical Testing of a 3D-printed L5 Vertebral Body Model

**DOI:** 10.7759/cureus.3893

**Published:** 2019-01-15

**Authors:** Michael A Bohl, Clinton D Morgan, Michael A Mooney, Garrett J Repp, Jennifer N Lehrman, Brian P Kelly, Steve W Chang, Jay D Turner, U. Kumar Kakarla

**Affiliations:** 1 Department of Neurosurgery, Barrow Neurological Institute, St. Joseph's Hospital and Medical Center, Phoenix, USA; 2 Department of Neurology, Barrow Neurological Institute, St. Joseph's Hospital and Medical Center, Phoenix, USA

**Keywords:** 3d printing, bone mineral density, pedicle screw, spine biomechanics, synthetic bone model

## Abstract

Background

We examined the biomechanical performance of a three-dimensional (3D)-printed vertebra on pedicle screw insertional torque (IT), axial pullout (APO), and stiffness (ST) testing.

Materials and methods

Seventy-three anatomically identical L5 vertebral body models (146 pedicles) were printed and tested for IT, APO, and ST using single-threaded pedicle screws of equivalent diameter (6.5 mm), length (40.0 mm), and thread pitch (2.6 mm). Print properties (material, cortical thickness [number of shells], cancellous density [in-fill], in-fill pattern, print orientation) varied among models. One-way analysis of variance was performed to evaluate the effects of variables on outcomes.

Results

The type of material significantly affected IT, APO, and ST (P < 0.001, all comparisons). For acrylonitrile butadiene styrene (ABS) models, in-fill density (25-35%) had a positive linear association with APO (P = 0.002), ST (P = 0.008), and IT (P = 0.10); similarly for the polylactic acid (PLA) models, APO (P = 0.001), IT (P < 0.001), and ST (P = 0.14). For the nylon material type, in-fill density did not affect any tested parameter. For a given in-fill density, material, and print orientation, the in-fill pattern significantly affected IT (P = 0.002) and APO (P = 0.03) but not ST (P = 0.23). Print orientation also significantly affected IT (P < 0.001), APO (P < 0.001), and ST (P = 0.002).

Conclusions

3D-printed vertebral body models with specific print parameters can be designed to perform analogously to human bone on pedicle screw tests of IT, APO, and ST. Altering the material, in-fill density, in-fill pattern, and print orientation of synthetic vertebral body models could reliably produce a model that mimics bone of a specific bone mineral density.

## Introduction

The Barrow Biomimetic Spine project aims to create a 3-dimensional (3D)-printed, synthetic spine model with high anatomical and biomechanical fidelity compared to that of a human cadaveric spine. The authors previously validated the radiographic performance of this synthetic spine model in L3-L5 segments [1]. For a synthetic spine model to adequately mimic cadaveric tissue, the quality of synthetic bone must perform similarly to human tissue on biomechanical testing.

Synthetic bone models are important tools for performing biomechanical analyses of various spinal instrumentation devices and techniques, and numerous synthetic bone models are commercially available [2, 3]. Polyurethane foam is a commonly used synthetic bone model, and it has been widely reported as a reasonable substitute for cadaveric bone [4-8]. Existing synthetic models such as polyurethane are regulated and standardized per guidelines of the American Society for Testing and Materials (ASTM-1839-08), with various grades of foam correlating to specific bone mineral densities (BMDs) [9]. Advantages of using a synthetic bone model rather than cadaveric bone for spinal biomechanical testing include reduced interspecimen variability, reduced cost, longer shelf life, ability to model different pathologic bone states using different foam grades, and elimination of institutional requirements for handling and testing human tissue [10].

A 3D-printed spine model must include a synthetic bone material that mimics human bone in both its corticocancellous architecture and its biomechanical performance on screw insertional torque (IT), axial pullout (APO) force, and stiffness (ST) testing. The model must furthermore demonstrate expected changes in these biomechanical performance measures when printed to mimic human bone of higher or lower BMD. The purpose of this study was to examine the biomechanical performance of a novel 3D-printed L5 vertebral body on IT, APO, and ST testing, and to validate this model against previously published data that define these outcome measures for cadaveric and living human bone at various BMDs.

## Materials and methods

Study material

A high-resolution computed tomography (CT) of a normal lumbar spine was segmented and converted into a 3D file using Materialise Mimics software (Materialise, NV, Leuven, Belgium). The complete L5 vertebra was extracted from this 3D file and converted to a stereolithography (.stl) file format. The .stl file was imported into the Simplify3D software package (Simplify3D, LLC, Blue Ash, Ohio, USA). Models were then printed using a FlashForge Creator Pro (FlashForge Corp., Zhejiang, China).

The models used in this study were printed using three different materials: acrylonitrile butadiene styrene (ABS), polylactic acid (PLA), and nylon. ABS is a common thermoplastic polymer that is petroleum-based and known for its impact resistance and durability. PLA is a biodegradable and bioactive thermoplastic derived from sugar-based substances (e.g., cornstarch, sugarcane, cassava root). PLA has a much lower glass transition temperature than ABS and is more brittle but also has higher impact resistance and toughness. Nylon is a family of thermoplastic synthetic polymers. The specific type of nylon used in this study is called Nylon 230 (Taulman 3D, LLC, Saint Peters, Missouri, USA), because it has a much lower glass transition temperature (230°C) than other types. 3D-printed nylon is known for its high durability, strength, and versatility in that thin layers of printed nylon remain very flexible whereas thick layers become rigid and stiff.

**Figure 1:**
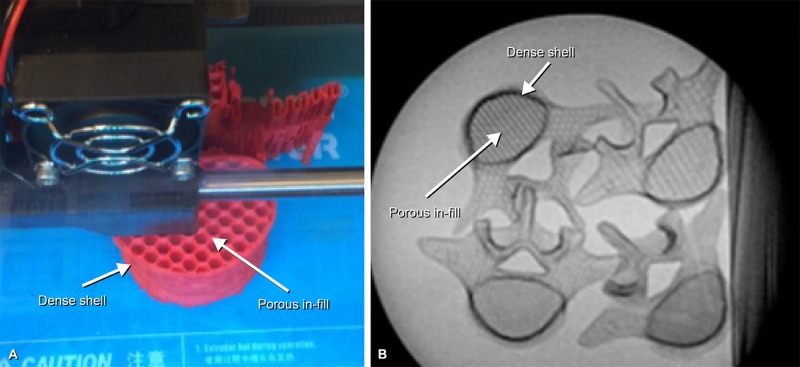
L5 vertebral body model being printed. (A) Photograph of an L5 vertebral body model printing. Arrows denote the dense shell layers and less dense in-fill. (B) Axial view of L5 vertebral body models under standard fluoroscopy. These views demonstrate the corticocancellous structure of the models, which is analogous in fluoroscopic appearance to that of human bone. *Used with permission from Barrow Neurological Institute, Phoenix, Arizona.*

Other evaluated print setting variables included the print shell, in-fill percent, in-fill pattern, and print orientation. The 3D-printed L5 vertebral body models are printed with a dense outer layer of plastic (the “shell”) and a much less dense inner component (the “in-fill”), analogous to the cortical and cancellous structure of human bone, respectively. Figure 1 demonstrates the shell and in-fill of a vertebral body model (Figure 1A) and how this structure mimics the corticocancellous architecture of human bone when viewed under fluoroscopy (Figure 1B). Both the shell and the in-fill can be modified to print at various thicknesses and densities. The in-fill can furthermore be modified to be printed in one of several different patterns, including hexagonal, diamond, and linear.

A number of printer settings were held constant for all models printed with a specific material. For the ABS models, the print temperature was held at 240°C, the print bed temperature at 110°C, the print resolution at 0.2 mm, and the print speed at 60 mm/s. For PLA, the print temperature was held at 230°C, the print bed temperature at 30°C, the print resolution at 0.2 mm, and the print speed at 60 mm/s. For nylon, the print temperature was held at 230°C, the print bed temperature at 50°C, the print resolution at 0.2 mm, and the print speed at 30 mm/s. These printer settings were not tested for their effect on the biomechanical performance of the model; they were kept constant across all models printed with the same material to avoid any error introduced by variation in these settings.

Historical results for comparison

To validate the vertebral body model’s utility as a synthetic bone substitute in biomechanical testing, we referenced historical data on cadaveric and living bone [10-15]. In 2013, Brasiliense et al. [11] compared the performance of a single-threaded vs. a dual-threaded screw on IT, APO, and ST testing. The same method was used to test the L5 synthetic vertebra model, using single-threaded screws of equivalent diameter (6.5 mm), length (40.0 mm), and thread pitch (2.6 mm). Screw insertion, IT, APO, and ST testing were all performed equivalently to that described by Brasiliense et al. to permit a meaningful comparison of the results they generated using cadaveric bone with the results generated in this study using the synthetic L5 vertebra models. All equipment used in our study during IT, APO, and ST testing was the same equipment used by Brasiliense et al., as these studies took place in the same laboratory.

Study design

**Figure 2:**
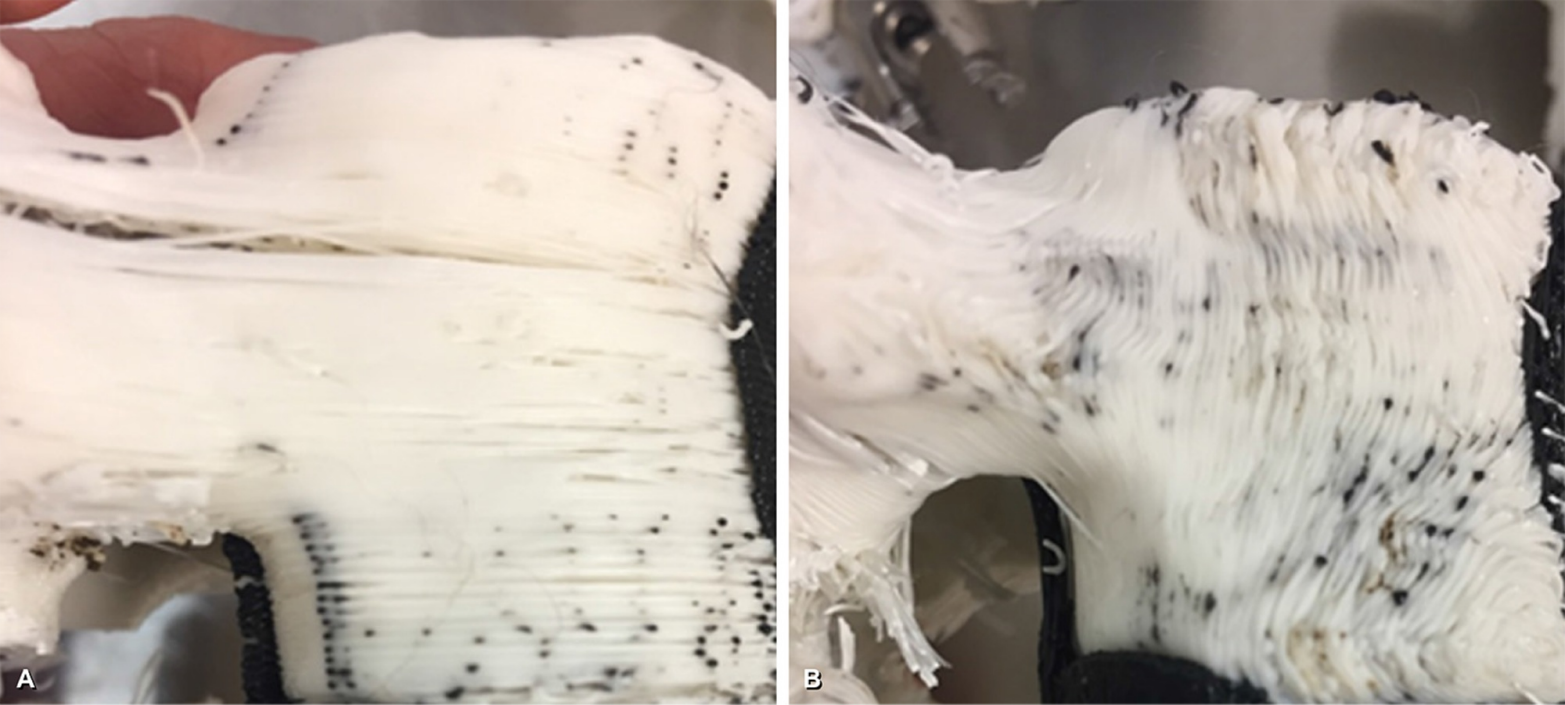
Model printed with a horizontal orientation. (A) Photograph of a model printed with a horizontal orientation. Note the layers of the plastic comprising the model are layered parallel to the endplates. (B) Photograph of a model printed with a vertical orientation. Note the layers of the plastic comprising the model are layered perpendicular to the endplates.* Used with permission from Barrow Neurological Institute, Phoenix, Arizona.*

Seventy-three L5 vertebral body models (146 pedicles) were printed from the same .stl file such that all the models were anatomically identical. ABS, PLA, and nylon models were printed with a shell density of 1-8 layers, and an in-fill density of 10-50%. Models were also printed with different in-fill patterns (hexagonal vs. linear vs. diamond), and different orientations on the print bed (horizontal vs. vertical print alignment). Figure 2A and Figure 2B demonstrate the difference between models printed with horizontal print alignment and vertical print alignment. Horizontal and vertical refer to the z-axis of the 3D printer in relation to the anatomical top and bottom of the L5 vertebra. When the model is printed in the horizontal orientation (Figure 2A), layers of plastic filament are placed on top of each other from the bottom to the top of the vertebral model. In the vertical orientation (Figure 2B), filament layers are parallel to the top and bottom of the vertebra and are stacked from the ventral vertebra to the dorsal vertebra.

Each model underwent pedicle screw insertion of the bilateral pedicles using a 6.5 × 40.0-mm screw with a single thread pitch of 2.6 mm. The same author (M.A.B.) inserted all pedicle screws to minimize differences in pedicle screw trajectory between models. To avoid bias, this author was blinded to the torque values. During pedicle screw insertion, a torque sensor measured and collected the IT at a rate of 5 Hz. After bilateral pedicle screws were inserted in the models, they were placed in a metal fixture and potted in a casting mold of SmoothCast 300Q resin (Smooth-On, Macungie, PA, USA).

**Figure 3:**
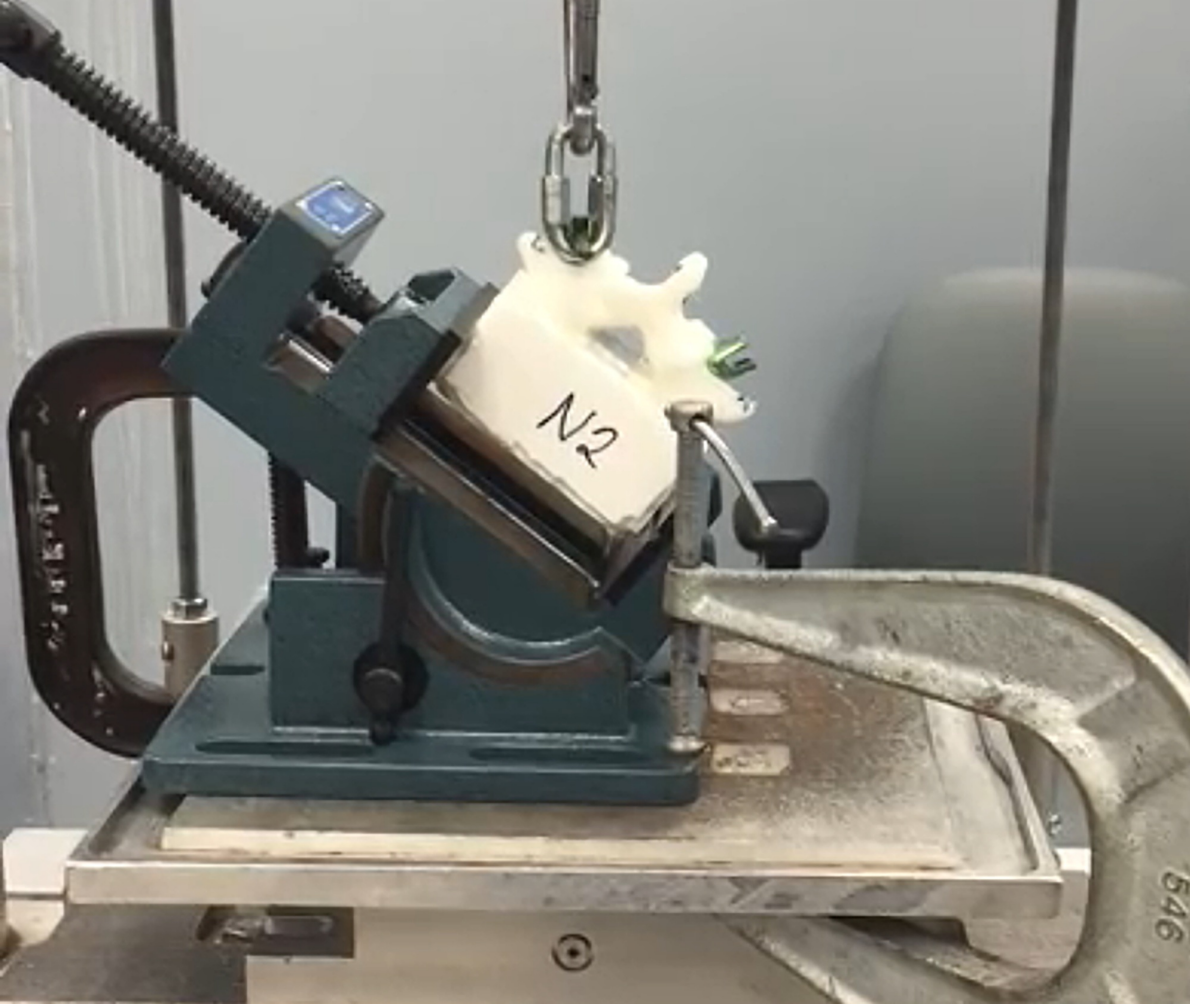
Vertebral body model in the angled vise grip during axial pullout (APO) testing. Photograph of a vertebral body model in the angled vise grip during APO testing. The vise grip is arranged to provide a true axial force on the pedicle screw.* Used with permission from Barrow Neurological Institute, Phoenix, Arizona.*

After the vertebral bodies were potted, a uniaxial servohydraulic test frame (858 Mini Bionix, MTS Test Systems Corp., Eden Prairie, Minnesota, USA) was used to conduct APO testing of each pedicle screw. In summary, an angle vise was used to affix the resin mold of each model to the base of the testing apparatus. The long axis of the pedicle screw to be tested was then aligned parallel to the axis of the testing apparatus to create a purely axial force vector on each pedicle screw. APO loading force was at a 10 mm/min displacement rate. Load versus displacement data were continuously recorded at a 10-Hz frequency until total screw failure, which was defined as the point on the load-displacement curve at which a precipitous decline occurs. APO was then calculated as the greatest load before failure. The load-displacement curve was then used to calculate the screw ST, defined as the steepest slope on the load-displacement curve. Figure 3 demonstrates a vertebral body model undergoing an APO test.

Statistical analysis

Descriptive statistics, including means and SDs, were collected for all models. The D’Agostino-Pearson normality test was used to determine the normalcy of the data. Left and right pedicles were compared separately and together. One-way analysis of variance (ANOVA) was performed to evaluate for the effect of material, shell density, in-fill density, in-fill pattern, and print pattern on measured outcomes.

## Results

Thirty-seven ABS models were printed and underwent complete testing. These models had shell density ranging from one to eight layers, in-fill density ranging from 10 to 50%, three different in-fill patterns (hexagonal, linear, diamond), and both horizontal and vertical print orientations. Twenty-seven PLA models and 27 nylon models were printed, all with a shell density of four or eight layers and an in-fill density of 25%, 30%, or 35%. See Table 1 for a summary of the successfully printed and tested L5 vertebral body models, their respective print settings, and the mean (SD) IT, APO, and ST values (Nm, N, and N/mm, respectively) for the model groups.

**Table 1:** 3D-printed vertebral models and outcomes summary*. ABS: Acrylonitrile butadiene styrene; APO: Axial pullout; IT: Insertional torque; NA: Not available; PLA: Polylactic acid; ST: Stiffness; 3D: Three-dimensional. *Values are mean (SD) unless indicated otherwise.

Model No.	N (No. pedicles)	Material	Shells, No.	In-fill Density, %	In-fill Pattern	Print Orientation	IT (Nm)	APO (N)	ST (N/mm)
1	2 (4)	ABS	1	10	Hexagonal	Horizontal	NA	382 (47)	95 (48)
2	2 (4)	ABS	1	50	Hexagonal	Horizontal	NA	1326 (303)	180 (84)
3	2 (4)	ABS	4	10	Hexagonal	Horizontal	NA	298 (52)	94 (36)
4	2 (4)	ABS	2	50	Hexagonal	Horizontal	NA	1463 (258)	275 (49)
5	2 (4)	ABS	4	50	Hexagonal	Horizontal	NA	1838 (272)	205 (85)
6	3 (6)	ABS	4	25	Hexagonal	Horizontal	0.878 (0.073)	852 (32)	707 (35)
7	3 (6)	ABS	4	30	Hexagonal	Horizontal	1.013 (0.037)	1133 (89)	795 (122)
8	3 (6)	ABS	4	35	Hexagonal	Horizontal	0.953 (0.004)	1194 (51)	736 (441)
9	3 (6)	ABS	8	25	Hexagonal	Horizontal	0.889 (0.033)	856 (174)	699 (94)
10	3 (6)	ABS	8	30	Hexagonal	Horizontal	0.917 (0.140)	1241 (111)	890 (105)
11	3 (6)	ABS	8	35	Hexagonal	Horizontal	1.099 (0.066)	1349 (88)	967 (31)
12	3 (6)	PLA	4	25	Hexagonal	Horizontal	1.157 (0.114)	1778 (444)	913 (242)
13	3 (6)	PLA	4	30	Hexagonal	Horizontal	1.303 (0.064)	2459 (91)	1046 (110)
14	3 (6)	PLA	4	35	Hexagonal	Horizontal	1.612 (0.023)	3069 (247)	1016 (125)
15	3 (6)	PLA	8	25	Hexagonal	Horizontal	1.012 (0.450)	2155 (350)	996 (216)
16	3 (6)	PLA	8	30	Hexagonal	Horizontal	1.388 (0.180)	3148 (615)	1157 (74)
17	3 (6)	PLA	8	35	Hexagonal	Horizontal	1.645 (0.072)	3674 (405)	1080 (217)
18	3 (6)	Nylon	4	25	Hexagonal	Horizontal	0.300 (0.063)	389 (389)	340 (286)
19	3 (6)	Nylon	4	30	Hexagonal	Horizontal	0.476 (0.126)	160 (26)	110 (13)
20	3 (6)	Nylon	4	35	Hexagonal	Horizontal	0.534 (0.021)	296 (53)	265 (34)
21	3 (6)	Nylon	8	25	Hexagonal	Horizontal	0.490 (0.082)	182 (39)	134 (49)
22	3 (6)	Nylon	8	30	Hexagonal	Horizontal	0.473 (0.046)	146 (12)	100 (32)
23	3 (6)	Nylon	8	35	Hexagonal	Horizontal	0.448 (0.164)	170 (26)	144 (68)
24	3 (6)	ABS	4	25	Linear	Horizontal	0.639 (0.148)	803 (357)	624 (196)
25	3 (6)	ABS	4	25	Diamond	Horizontal	0.928 (0.132)	1180 (220)	779 (161)
26	3 (6)	ABS	4	25	Hexagonal	Vertical	0.617 (0.104)	467 (21)	517 (104)

IT, APO, and ST tested values were normally distributed (D’Agostino-Pearson normality test, P > 0.05 for all). In the analysis of all tested variables from all different material types, shells, in-fills, in-fill patterns, and orientations, no significant variance was found between pedicles on the left versus the right side for IT, APO, and ST (P > 0.05 for all).

**Figure 4:**
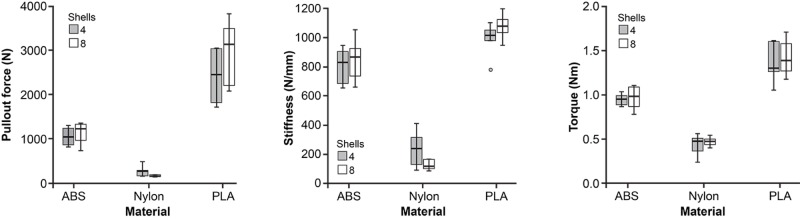
Box plot summaries demonstrating the effect of material type on the tested parameters. Box plot summaries demonstrating the effect of material type on the tested parameters. (Left) Effect of material type on APO; (Middle) effect of material type on ST; (Right) effect of material type on IT. ABS: Acrylonitrile butadiene styrene; PLA: Polylactic acid. *Used with permission from Barrow Neurological Institute, Phoenix, Arizona.*

**Figure 5:**
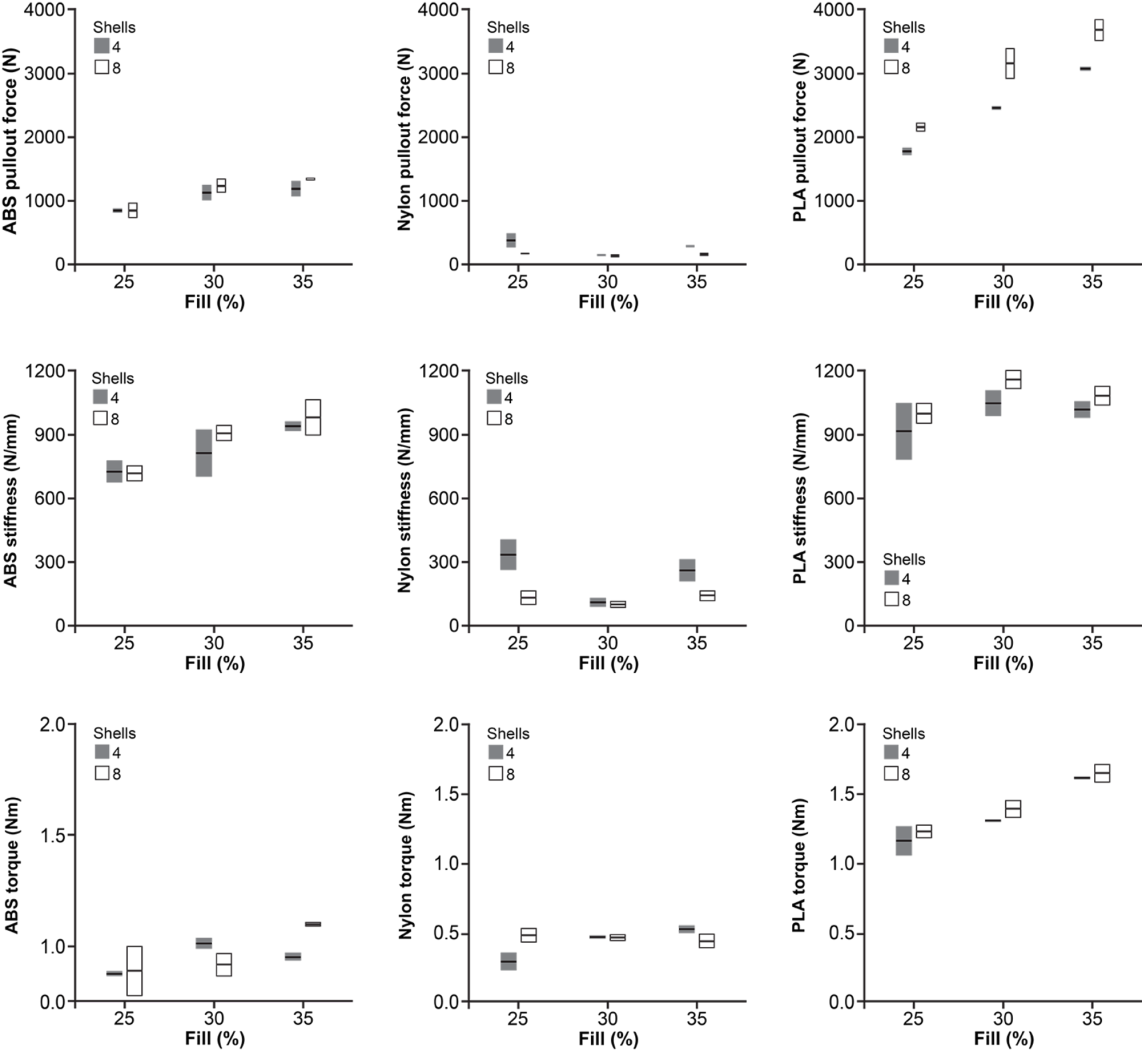
Box plot summaries demonstrating the effect of in-fill on the tested parameters. Box plot summaries demonstrating the effect of in-fill on the tested parameters. (Top row) Effect of in-fill on APO for ABS, nylon, and PLA models; (Middle row) effect of in-fill on ST for ABS, nylon, and PLA models; (Bottom row) effect of in-fill on IT for ABS, nylon, and PLA models. ABS: Acrylonitrile butadiene styrene; PLA: Polylactic acid.* Used with permission from Barrow Neurological Institute, Phoenix, Arizona.*

The type of material significantly affected IT, APO, and ST (P < 0.001 for all comparisons, Table 2). Figure 4 provides a box plot summary of the effect of material type on the tested parameters. PLA demonstrated the highest IT, APO, and ST values, followed by ABS and nylon, respectively. For the ABS models, in-fill density (25-35%) had a positive linear association with APO (P = 0.002), ST (P = 0.008), and IT (P = 0.10). For the PLA models, APO (P = 0.001), IT (P < 0.001), and ST (P = 0.14) had a similarly positive linear association with in-fill density. For the nylon material type, in-fill density did not affect any tested parameter. Figure 5 provides a box plot summary of the effect of in-fill on the tested parameters for models of all three material types.

**Table 2:** Comparisons of material, in-fill, and shell*. ABS: Acrylonitrile butadiene styrene; APO: Axial pullout; IT: Insertional torque; PLA: Polylactic acid; ST: Stiffness. *Values are mean (SD) unless indicated otherwise.

Variable	IT (Nm)	APO (N)	ST (N/mm)
Material			
ABS (n = 12)	0.96 (0.10)	1104 (218)	830 (128)
Nylon (n = 12)	0.45 (0.09)	223 (103)	182 (102)
PLA (n = 12)	1.39 (0.20)	2713 (684)	1034 (106)
P value	.001	.001	.001
Material percent fill			
ABS			
ABS 25 (n = 4)	0.88 (0.09)	854 (95)	702 (52)
ABS 30 (n = 4)	0.97 (0.07)	1187 (139)	843 (110)
ABS 35 (n = 4)	1.03 (0.09)	1271 (129)	946 (76)
P value	.10	.002	.008
Nylon			
Nylon 25 (n = 4)	0.40 (0.13)	285 (146	237 (135)
Nylon 30 (n = 4)	0.47 (0.02)	152 (16)	105 (20)
Nylon 35 (n = 4)	0.49 (0.07)	233 (75)	204 (83)
P value	.28	.20	.17
PLA			
PLA 25 (n = 4)	1.19 (0.10)	1966 (226)	954 (124)
PLA 30 (n = 4)	1.35 (0.07)	2803 (442)	1101 (86)
PLA 35 (n = 4)	1.63 (0.06)	3371 (374)	1047 (59)
P value	.001	.001	.14
Material thickness			
ABS			
ABS 4 (n = 6)	0.95 (0.06)	1060 (193)	808 (125)
ABS 8 (n = 6)	0.97 (0.13)	1148 (250)	852 (139)
P value	.73	.51	.58
Nylon			
Nylon 4 (n = 6)	0.44 (0.12)	281 (122)	238 (119)
Nylon 8 (n = 6)	0.47 (0.05)	166 (24)	126 (33)
P value	.53	.047	.07
PLA			
PLA 4 (n = 6)	1.36 (0.22)	2435 (578)	.991 (112)
PLA 8 (n = 6)	1.42 (0.20)	2992 (714)	1077 (87)
P value	.62	.17	.17

For a given in-fill density, material, and print orientation, the in-fill pattern had a significant effect on IT (P = 0.002) and APO (P = 0.03) but not on ST (P = 0.23). Print orientation also significantly affected IT (P < 0.001), APO (P < 0.001), and ST (P = 0.002). Shell density did not significantly affect the biomechanical performance of the synthetic bone models. See Table 3 for a summary of these results.

**Table 3:** Comparison of in-fill patterns and print orientations. ABS: Acrylonitrile butadiene styrene; ANOVA: Analysis of variance; APO: Axial pullout; IT: Insertional torque; ST: Stiffness.

Model No.	N	Material	Shells	In-fill Density, %	In-fill Pattern	Print Orientation	IT (Nm)	APO (N)	ST (N/mm)
6	3	ABS	4	25	Hexagonal	Horizontal	0.878 (0.073)	852 (32)	707 (35)
24	3	ABS	4	25	Linear	Horizontal	0.639 (0.148)	803 (357)	624 (196)
25	3	ABS	4	25	Diamond	Horizontal	0.928 (0.132)	1180 (220)	779 (161)
ANOVA							P = .002	P = .03	P = .23
6	3	ABS	4	25	Hexagonal	Horizontal	0.878 (0.073)	852 (32)	707 (35)
26	3	ABS	4	25	Hexagonal	Vertical	0.617 (0.104)	467 (21)	517 (104)
t-test							P < .001	P < .001	P = .002

## Discussion

Main results

ABS and PLA demonstrated good correlation between model in-fill density and biomechanical performance measures, and as such both are good candidate materials for a synthetic lumbar vertebral body model. In contrast, nylon does not appear to be a good material, as changes in the evaluated print parameters did not result in predictable changes in the tested outcomes. Interestingly, PLA models had significantly greater IT, APO, and ST values than ABS models. Anecdotally, however, the surgeon authors (M.A.B., C.D.M., M.A.M., S.W.C., J.D.T., U.K.K.) decided that the ABS models felt much more similar to human bone than the PLA models when cannulating pedicles and placing pedicle screws. Specifically, the PLA did not break or deform under the pressure of a pedicle-finding probe but rather became somewhat soft. This observation may be explained by the much lower glass transition temperature of PLA (60°C) compared to ABS (105°C); the friction generated by twisting a pedicle-finding probe or inserting a pedicle screw into the PLA model likely causes the model to deform locally rather than break. ABS would readily break when contacting a twisting pedicle probe, creating a feeling similar to that of human bone. Given that the ABS and PLA models performed with equivalent reliability in terms of their linear associations between print variables and tested outcomes, we believe that ABS is the most promising of these three materials for further development and use as a synthetic model of a lumbar vertebra.

Also significantly impacting the tested outcomes were in-fill pattern and print orientation. Interestingly, in-fill pattern predictably impacted all three tested outcomes, with the diamond pattern producing higher IT, APO, and ST values than the hexagonal and linear patterns. This finding will be important when selecting specific print parameters for creation of synthetic vertebral body models to be instrumented, as the choice of in-fill pattern will significantly impact the screw performance in those models. Similarly, the print orientation had a highly significant impact on tested outcomes, although the direction of effect was different for IT than for APO and ST. This finding likely relates to the observation that the models tended to fail on APO testing in a plane parallel to the print orientation. The IT was measured during screw insertion, whereas the APO and ST were measured during screw pullout. The impact of the print orientation is therefore likely to impact the tested outcomes differently during these tests.

For ABS models, in-fill had a significant effect on IT and APO but not on ST. Similarly, in-fill pattern significantly affected IT and APO but not ST. However, ST was significantly different among vertebral body models of different material. Perhaps this finding indicates that ST is more affected by material type than the other tested outcomes.

Comparison to historical data

In 2013, Brasiliense et al. [11] analyzed the IT, APO, and ST of 17 cadaveric vertebral bodies using the same screw size, screw thread pitch, and testing equipment as in this study. The mean BMD of the 17 specimens in that reference study was 0.794 (0.147) g/cm^2^ (range 0.557-1.071 g/cm^2^), meaning the tested specimens were, on average, osteopenic. Of the 17 tested vertebrae, 10 were osteoporotic, two were osteopenic, and all the models subjected to APO testing were below the cutoff for normal BMD. The mean (SD) IT, APO, and ST of the cadaveric vertebral bodies tested by Brasiliense et al. [11] were 0.69 (0.54) Nm, 1002 (502) N, and 426 (194) N/mm, respectively. Comparing these values to the synthetic models we tested, we find that the ABS models printed with an in-fill density of 25% are most similar in IT (0.88 vs. 0.69 Nm) and APO (854 vs. 1002 N) to the cadaveric vertebral bodies, but demonstrate 1.6 times the ST (702 vs. 426 N/mm). It is also worth noting that the ABS models in aggregate demonstrated an SD much lower than the SDs reported by Brasiliense et al. [11] for the vertebral bodies (IT 0.10 vs. 0.54 Nm, APO 218 vs. 502 N, and ST 128 vs. 194 N/mm). Brasiliense et al. [11] also measured the IT and APO of the same screws in high-porosity and low-porosity foams. These foams produced SDs similar to those of the ABS 25% models for IT (0.09 vs. 0.31 high-porosity or 0.73 low-porosity) and APO (95 vs. 65 high-porosity or 280 low-porosity). These results support our claims that the synthetic vertebral bodies may be a superior testing platform to cadaveric specimens as they contain less interspecimen variability, which should therefore result in better data with fewer models needed for comparison studies.

By using the linear regression analysis correlating APO and BMD that was published in 1994 by Halvorson et al. [12], we can predict the BMD we are likely to mimic with certain model materials and print settings. Nylon, for example, had a mean (SD) APO force of 223 (103) N; using the Halvorson et al. linear regression, this value correlates with a BMD <0.6 g/cm^2^. A BMD value this low represents extreme osteoporosis and falls off the normal curve entirely. But the mean APO force for ABS (1104 {218} N) and PLA (2713 {684} N) models would correlate with a BMD of approximately 1.0 g/cm^2^ and >1.4 g/cm^2^, respectively. The same type of comparisons to historical data can be performed for IT and ST. Previous studies correlating BMD with IT and ST show that the studied synthetic model produces IT and ST values similar to those described in these historical data and that these variables can be reliably predicted through changes in model material, in-fill density, and in-fill pattern [12-15]. Thus, it is easy to imagine the studied synthetic models being printed to perform analogously on IT, APO, and ST to human bone of a specific BMD. These models have potential, therefore, to become promising new platforms for spine biomechanics research. Furthermore, this study validates their continued use as synthetic bone in our continued efforts to 3D print a synthetic spine model with high anatomical, radiographic, and biomechanical fidelity to human tissue.

Limitations

This study has several limitations that must be considered when interpreting the results. Small differences in screw insertion technique, screw size or design, or data analysis not captured in the historical comparison data may have substantially altered results. Also, our statistical comparison groups included three vertebral bodies (six pedicles) printed with each specific set of print parameters. Printing more models would have increased the power of the study to detect the effect of print settings on outcomes. Finally, small differences between pedicle screw trajectories in each model may have increased the variability of results. We attempted to control for this variable by having a single surgeon insert all pedicle screws in the study.

## Conclusions

The 3D-printed vertebral body models made of ABS and PLA performed analogously to human bone on pedicle screw tests of IT, APO, and ST. By altering the material, in-fill density, in-fill pattern, and print orientation of the synthetic vertebral body models, one could reliably produce a model that mimics bone with a specific BMD. As such, these synthetic models represent a promising new tool in spine biomechanics research, and they have promising potential utility for surgical planning and surgical education.
